# Helminth Infections in Cattle and Goats in Kanchanaburi, Thailand, with Focus on Strongyle Nematode Infections

**DOI:** 10.3390/vetsci8120324

**Published:** 2021-12-12

**Authors:** Nicharee Income, Jarinee Tongshoob, Sarawut Taksinoros, Poom Adisakwattana, Chawarat Rotejanaprasert, Pannamas Maneekan, Nathamon Kosoltanapiwat

**Affiliations:** 1 Department of Microbiology and Immunology, Faculty of Tropical Medicine, Mahidol University, 420/6 Ratchawithi Road, Ratchathewi, Bangkok 10400, Thailand; nicharee.inc@mahidol.ac.th (N.I.); jarinee.pan@mahidol.ac.th (J.T.); 2 Livestock and Wildlife Hospital, Mahidol University, 199 Lum Sum, Sai Yok, Kanchanaburi 71150, Thailand; 3 Department of Clinical Science and Public Health, Faculty of Veterinary Science, Mahidol University, 999 Salaya, Phutthamonthon, Nakhon Pathom 73170, Thailand; sarawut.tak@mahidol.ac.th; 4 Department of Helminthology, Faculty of Tropical Medicine, Mahidol University, 420/6 Ratchawithi Road, Ratchathewi, Bangkok 10400, Thailand; poom.adi@mahidol.ac.th; 5 Department of Tropical Hygiene, Faculty of Tropical Medicine, Mahidol University, 420/6 Ratchawithi Road, Ratchathewi, Bangkok 10400, Thailand; chawarat.rot@mahidol.ac.th (C.R.); pannamas.man@mahidol.ac.th (P.M.); 6 Mahidol-Oxford Tropical Medicine Research Unit, Faculty of Tropical Medicine, Mahidol University, 420/6 Ratchawithi Road, Ratchathewi, Bangkok 10400, Thailand

**Keywords:** cattle, goat, helminth infection, strongyle nematode, risk factor, PCR

## Abstract

Gastrointestinal helminths are major enteric parasites affecting the health of important livestock ruminants, such as cattle and goats. It is important to routinely survey these animals for helminth infections to allow effective management and control programs to be implemented. A cross-sectional helminth survey carried out in Kanchanaburi Province, Thailand, revealed the infection rate of gastrointestinal helminths in cattle (*n* = 157) and goats (*n* = 117) to be 35.7% and 88%, respectively, by microscopic fecal examination, and a 100% herd prevalence was observed in goats. Eggs of strongyle nematodes, *Strongyloides* spp., *Trichuris* spp., *Capillaria* spp., *Paramphistomum* spp., and *Moniezia* spp. were detected, with a relatively high rate of strongyle nematode infection in both cattle (28.7%) and goats (86.3%). Mixed infections were observed in 14.3% and 35.9% of egg-positive samples from cattle and goats, respectively. Risk factor analysis showed that dairy cattle were 5.1 times more likely to be infected with strongyles than meat cattle. In contrast, meat goats were 9.3 times more likely to be infected with strongyles than dairy goats. The inverse findings in cattle and goats are discussed. Female gender was associated with a higher risk of strongyle infection in goats. DNA sequencing and in-house semi-nested PCR with primers specific to a region in the internal transcribed spacer 2 (ITS2) were successfully used to identify strongyle genera in randomly selected egg-positive cattle (*n* = 24) and goat (*n* = 24) samples. Four strongyle genera, i.e., *Cooperia* spp., *Haemonchus* spp., *Oesophagostomum* spp., and *Trichostrongylus* spp. were identified by DNA sequencing. By semi-nested PCR, *Cooperia* spp. were detected as a major parasite of cattle (70.8%), whereas *Haemonchus* spp. were abundant in goats (100%). The majority of samples from cattle (58.3%) and goats (95.8%) were found to coinfect with at least two strongyle genera, suggesting that coinfection with multiple strongyle genera was more common than single infection in these animals.

## 1. Introduction

Gastrointestinal helminths are one of the most important disease-causing agents in veterinary medicine, especially in livestock, and lead to economic losses as a result of a decrease in meat, milk, or wool production [1,2]. Most gastrointestinal helminths infect animals via the ingestion of infective-stage larvae or eggs. The eggs and larvae are excreted with the host’s feces into the environment and become a source of transmission. Strongyle nematodes of the order *Strongylida* are an important group of gastrointestinal helminths that significantly affect the health of ruminants, especially in tropical areas [3,4,5].

In Thailand, a prevalence of strongyle infections (27%) was reported in beef cattle from Nan Province in 2005 [6]. Those cattle were raised in a native pasture grazing system with poor sanitation and a lack of deworming. A high strongyle infection rate (79.47%), with 100% herd prevalence, was found among goats in Nakhon Pathom Province, Thailand, in 2010 [7]. The study showed that the housing system, deworming interval, and type of goats in the herd were risk factors for intestinal parasite infection. Surveys conducted in other Southeast Asian countries also point to the importance of strongyle infections in ruminants. In Lao PDR, strongyles were detected in 36% of cattle and 93% of goat fecal samples collected in 2010 [3]. *Trichostrongylus* spp., *Haemonchus* spp., and *Oesophagostomum* spp. were reported in cattle and goats, while *Cooperia* spp. were identified in cattle. *Trichostrongylus* spp. and *Haemonchus* spp. were found to be dominant parasites in cattle and goats, respectively. A report from Malaysia in 2014 described the incidence of strongyle infection in cattle (11.2%), and goats (63.1%) [4]. In the same study, a coinfection of two strongyles, *Haemonchus* spp. and *Trichostrongylus* spp., was detected in goat feces by amplification and sequencing of the internal transcribed spacer 2 (ITS2) DNA region. Furthermore, 100% herd prevalence and 96.22% animal-level prevalence of strongyles were reported in goats raised in the Philippines in 2016 [8], and the strongyle genera detected were *Haemonchus* spp., *Trichostrongylus* spp., *Oesophagostomum* spp., *Cooperia* spp., and *Chabertia* spp. Evidence of strongyle nematode infections in ruminants has been consistently found in South Asia, Africa, and Europe until the present day [9,10,11,12,13]. Among these, anthelmintic drug resistance was widely reported in goats infected with *Haemonchus* spp. [12,13]. For decades, benzimidazoles such as albendazole, imidothiazoles-tetrahydropyrimidines, and avermectin have been the major anthelmintic classes used for treating gastrointestinal nematodes of small ruminants. However, recent reports have shown a growing resistance to these drugs both in vitro and in vivo [12,13,14,15]. Factors contributing to the development of drug resistance could be mixed grazing of difference animal species such as goats and sheep, using under-dosage of anthelmintics, and using the same group of drugs for a long time [15,16]. Besides strongyles, other nematodes, such as *Trichuris* spp., as well as cestodes, such as *Moniezia* spp., and rumen flukes (trematodes), such as *Paramphistomum* spp., have been found to cause less severe problems in ruminants. Eggs of *Moniezia* spp. were detected in the feces of cattle, goats, and sheep in Vietnam [17] and goats in Thailand [7], and rumen fluke have been reported to infect beef cattle in Thailand, with no seriously observable clinical signs [6].

For diagnosis of veterinary helminth infections in Thailand, a microscopic stool examination is routinely performed. The technique provides a wide range of parasite detection but barely identifies the parasite at genus and species level. Molecular identification by PCR is considered to be a rapid, uncomplicated, and cost-effective method with high sensitivity and specificity that has enabled the accurate identification of parasite species [4,17]. The genomic regions widely used in the molecular detection and identification of the parasitic helminths are ITS of ribosomal DNA (rDNA), which in eukaryotes features non-transcribed spacer (NTS), external transcribed spacer (ETS), 18S rDNA, ITS1, 5.8S rDNA, ITS2, and 28S rDNA [4,17,18]. Coding regions of rDNA are highly conserved among species and, hence, are appropriate for pan-strongyle primer design, whereas ITS regions are more variable because of insertions, deletions, and point mutations. The ITS2 sequence has been utilized for strongyle identification owing to its high intraspecific sequence homogeneity and high interspecific sequence divergence [4]. Analysis of ITS2 has previously been used to confirm morphological diagnoses of *Trichostrongylus* spp., *Oesophagostomum* spp., and *Haemonchus* spp. [3].

Due to a lack of published information concerning helminthic infections in cattle and goats in Kanchanaburi Province, Thailand, the prevalence of gastrointestinal helminthic infection in cattle and goats was surveyed by egg microscopic examination. As the animals were found to be infected with strongyle nematodes at a high rate, the strongyles subsequently became the main focus of the study. Risk factors associated with strongyle infection were investigated. ITS2 sequencing was used to identify strongyle genera that were circulating in cattle and goats. In-house semi-nested PCR was used to differentiate strongyles to the genus level and ascertain the coinfection status of animals.

## 2. Materials and Methods

### 2.1. Sample and Data Collection

**Figure 1:**
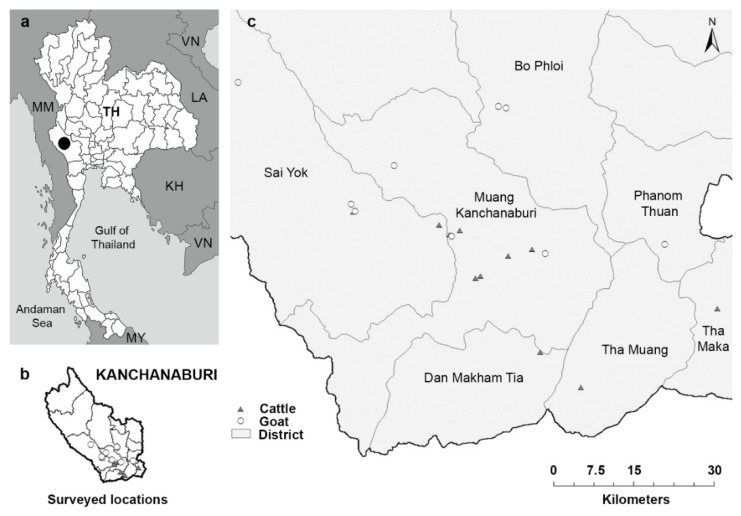
Map showing the sample collection sites in Kanchanaburi, Thailand. (**a**) Thailand map with Province outline—the black circle indicates Kanchanaburi Province. TH, Thailand; KH, Cambodia; LA, Lao People’s Democratic Republic; MM, Myanmar; MY, Malaysia; VN, Vietnam. (**b**,**c**) Surveyed locations in Kanchanaburi. Triangles represent the cattle farms (11 farms), and circles represent the goat farms (9 farms). The names of the districts in Kanchanaburi Province are shown on the map. Images in (**b**,**c**) were adapted from the authors’ published work. (Reprinted with permission from Income, N.; Kosoltanapiwat, N.; Taksinoros, S.; Leaungwutiwong, P.; Maneekan, P.; Chavez, I.F. (2019). Copyright © 2019 American Society for Microbiology, Appl. Environ. Microbiol. 85: e02420-18 doi: 10.1128/AEM.02420-18.).

Fecal and blood samples, and animal information were collected from 157 cattle and 117 goats in 11 cattle farms and 9 goat farms, respectively, located in seven districts of Kanchanaburi Province in the western part of Thailand, in May–July 2016 (Figure 1). A cross-sectional survey was performed, simultaneously collecting samples and relevant demographic and management data during a single farm visit. Random sampling was conducted in farms that allowed our visit. The cattle farms included 10 intensive management farms, where cattle were restricted to the farm area and fed concentrated food and roughage, such as grass, hay, or corn, and one extensive management farm, where cattle were released to graze freely during the day and were housed indoors at night. The goat farms included eight extensive management farms and one intensive management farm, where goats were housed in a cage and fed with concentrated food and roughage, including grass and leaves. Each farm housed 15 to 50 animals.

Fecal samples were directly collected from the animal rectum using clean gloves. Whole blood was collected from the tail vein (cattle) or jugular vein (goat), preserved in an EDTA tube, and sent to the Hematology Laboratory, Livestock and Wildlife Hospital, Faculty of Veterinary Science, Mahidol University, Kanchanaburi, within 24 h. The packed cell volume (PCV) was determined by microhematocrit centrifugation using the EDTA-whole blood and categorized as anemic (PCV < 22% in goat, PCV < 24% in cattle) or non-anemic (PCV ≥ 22% in goat, PCV ≥ 24% in cattle) [19].

Animal identification (name and number), gender, age, production purpose (dairy or meat), farm management (intensive or extensive management), and deworming interval were recorded. Animals were grouped as young (<1 year) and adult (1 year and above). Body condition (thin to obese) was scored for cattle [20] and goats [21]. Oral mucosa color was observed and recorded as pink, pale pink or pale. All examinations were performed by a trained veterinarian.

### 2.2. Microscopic Examination

Microscopic examination of fecal samples was conducted using simple flotation and formalin-ether concentration techniques.

Flotation technique: Approximately 2 g of feces were mixed with 10 mL of saturated NaCl and filtered through two layers of wet cotton gauze into a 15 mL conical tube. Additional saturated NaCl was added to fill the tube, leaving a convex meniscus at the top, then a coverslip was carefully placed over the opening of the test tube. After leaving the test tube to stand for 10–15 min, the coverslip was carefully and vertically removed from the tube, immediately placed on a microscope slide, and examined under a light microscope.

Formalin-ether concentration technique: Approximately 2 g of feces were mixed with 10 mL of 10% formalin and filtered through two layers of wet cotton gauze into a 15 mL conical tube. Additional 10% formalin was added to adjust to a total volume of 10 mL, followed by the addition of 2 mL of ether. The liquid was shaken vigorously and centrifuged at 400× *g* for 2 min. Four layers became visible: a top layer of ether, a second debris plug layer, a third clear layer of formalin, and a fourth layer of sediment. The debris plug was detached from the side of the tube using a small stick. The layers of ether, debris plug, and formalin were poured off to leave a small amount of formalin and sediment (approximately 2 mL). The tube was left undisturbed for 10 min to allow the precipitate to form. The residual liquid was removed by pipetting, leaving about 0.5 mL of formalin. The precipitate and formalin were mixed and dropped onto a slide, covered with a coverslip, and examined under a light microscope.

Helminth egg identification and semi-quantification were performed according to the parasitology laboratory manual used at the Livestock and Wildlife Hospital, Mahidol University, Thailand. Egg burdens were graded at levels of 1–4. Grade 1, 2, 3, and 4 refer to 1–4, 5–8, 9–12, and 13 or more eggs per slide, respectively. Samples with at least grade 1 egg burden were considered as a positive sample.

### 2.3. Fecal Sample Preparation and DNA Extraction

Approximately 2 g of feces were mixed with 10 mL of 2% dish-washing soap solution and filtered through two layers of wet cotton gauze into a 15-mL conical tube. The tube was centrifuged at 1500× *g* for 10 min, and the liquid was discarded. The pellet was resuspended in 10 mL of sterile water, and the steps of filtration, centrifugation, and pellet dissolution were repeated twice. After the three rounds of filtering and centrifugation, the fecal pellet was resuspended in 0.5 mL sterile water, transferred to a microcentrifuge tube, and centrifuged at 5500× *g* for 5 min. The liquid was totally removed by pipetting, and the pellet was stored at −80 °C for subsequent DNA extraction.

The processed fecal pellet was snap-frozen in liquid nitrogen and ground immediately using a disposable pestle for three cycles. Subsequently, the sample was vortex-mixed in 1.4 mL of buffer ASL provided in the QIAamp DNA stool mini kit (Qiagen, Hilden, Germany) and transferred into a 2-mL sample tube RB (Qiagen, cat. no. 990381). A sterile stainless-steel bead, 5 mm in diameter (Qiagen, cat. no. 69989), was added to the tube, and the sample was homogenized using TissueLyser LT (Qiagen) at 50 Hz for 5 min, twice. The homogenized sample was subjected to DNA extraction using the QIAamp DNA Stool Mini Kit according to the manufacturer’s instructions. DNA was eluted with 50 µL of AE elution buffer and stored at −20 °C.

### 2.4. Semi-Nested PCR for Detection and Differentiation of Strongyles

To detect the presence of strongyle helminths in the fecal samples, PCR was performed with primers specific to regions of strongyle nematode ribosomal DNA and ITS2 (Table 1). The PCR reaction contained 1 × MyTaq HS Red Mix (Bioline, London, UK), 0.2 µM of each forward (Strongyle F2) and reverse (Strongyle R3) primer, and 1 µL of DNA template in a total volume of 25 µL. The thermal cycling conditions were set as 95 °C for 3 min, followed by 35 cycles of 95 °C for 30 s, 50 °C for 30 s, and 72 °C for 30 s, and a final elongation at 72 °C for 10 min. PCR products were resolved and visualized by agarose gel electrophoresis, and sent for DNA sequencing.

In-house semi-nested PCR primers were used to detect and differentiate the four strongyle genera that were found in this study, i.e., *Cooperia* spp., *Haemonchus* spp., *Oesophagostomum* spp., and *Trichostrongylus* spp. Primer sequences and expected PCR product sizes are shown in Table 1. Alignments of strongyle sequences and the specificity of the primers are shown in the Supplemental Figures S1 and S2. The semi-nested PCR reaction and PCR thermal cycling conditions were set up as mentioned above, except that the reverse primer was replaced with the genus-specific primer and 1 µL of diluted F2/R3 PCR product (1:100 in nuclease-free water) was used as the template.

### 2.5. Nucleotide Sequencing and Phylogenetic Analysis

PCR products of the expected size were excised from an agarose gel. The DNA was purified using a QIAquick gel extraction kit (Qiagen) following the manufacturer’s instructions. Purified PCR products were sent for nucleotide sequencing (Bioneer, Daejeon, Korea) using F2 primers. Sequencing chromatograms were inspected and processed using BioEdit 7.0.4.1. Nucleotide sequences were queried against the NCBI database using the BLAST tool and aligned with published reference sequences using ClustalW in BioEdit. A total of 14 strongyle sequences were submitted to the GenBank database and received the accession numbers MT294426, MT294427, MT294428, MT294429, MT294430, MT294431, MT294432, MT294433, MT294434, MT294435, MT294436, MT294437, MT294438, and MT294439. A phylogenetic tree was constructed using the maximum likelihood method based on the Kimura 2-parameter model and bootstrap resampling analysis of 1000 replicates in MEGA 7.0.21.

### 2.6. Statistical Analysis

The infection rate of strongyles and corresponding 95% confidence intervals (CI) were calculated using the Wilson approximation method. The univariable analysis by Chi-squared test was performed to evaluate risk factors at a 5% level of significance (*p* < 0.05) using the status of strongyle infection as the dependent variable and the animal and management factors (gender, age, body condition score, mucous membrane, PCV categories, production purpose, farm management, deworming interval, and flocks) as the independent variables. Significant variables (*p* < 0.05) in the univariable analysis were used to perform a backward stepwise multivariable logistic regression model to calculate the adjusted odds ratios (AOR) at 5% level of significance. Data analyses were conducted using RStudio software version 1.4.1717.

## 3. Results

### 3.1. Detection of Gastrointestinal Helminth Eggs in Cattle and Goat Feces by Microscopic Examination

**Figure 2:**
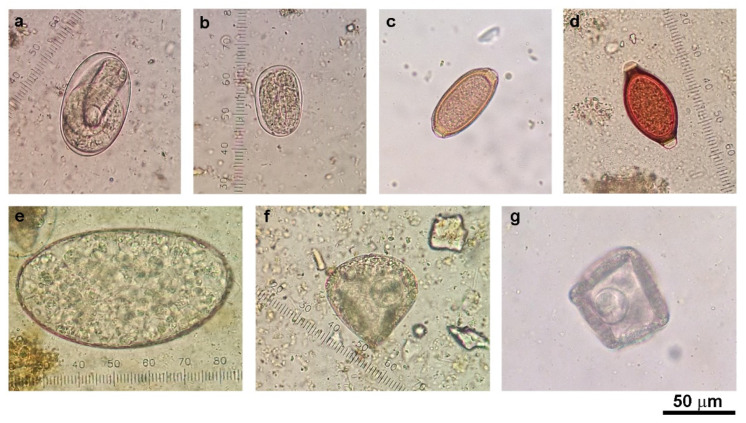
Morphology of gastrointestinal helminth eggs detected in cattle and goat feces by microscopic examination: (**a**) Strongyle egg (larvated egg); (**b**) *Strongyloides* spp. egg (larvated egg); (**c**) *Capillaria* spp. egg; (**d**) *Trichuris* spp. egg; (**e**) *Paramphistomum* spp. egg; (**f**) *Moniezia* spp. egg from goat; and (**g**) *Moniezia* spp. egg from cattle. Photos were taken under 400× magnification.

Fecal samples were collected from 11 cattle farms and 9 goat farms located in seven districts of Kanchanaburi Province, Thailand, in 2016 (Figure 1). The presence of helminth eggs in all cattle (*n* = 157) and goat (*n* = 117) fecal samples was qualitatively examined under a microscope after processing the samples with simple flotation and formalin-ether concentration techniques. The eggs of strongyle nematodes, *Strongyloides* spp., *Trichuris* spp., *Capillaria* spp., *Paramphistomum* spp., and *Moniezia* spp. were detected (Figure 2). Gastrointestinal helminth eggs were detected in 35.7% of cattle samples and 88% of goat samples. At the farm level, helminth egg-positive samples were found in 10 of the 11 cattle farms (90.9% herd prevalence) and in all nine goat farms (100% herd prevalence). The numbers and percentages of samples containing helminth eggs are shown in Table 2. Mixed infections of more than one type of helminth were found in 14.3% (8/56) and 35.9% (37/103) of egg-positive cattle and goat samples, respectively. Strongyle nematode eggs were most the abundant egg type in both cattle (28.7%) and goats (86.3%).

### 3.2. Infection Rate, Egg Burden and Risk Factors of Strongyle Infection among Cattle and Goats in Kanchanaburi Province

**Figure 3:**
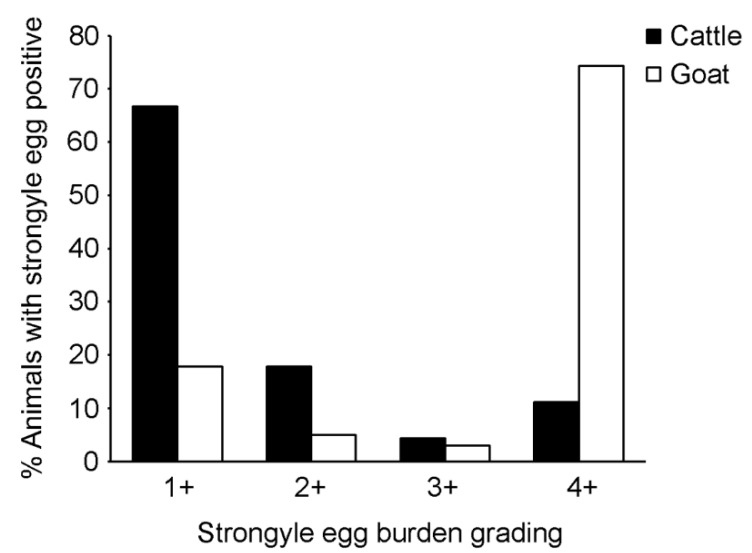
Strongyle egg burden grading. Strongyle eggs were observed and graded under a microscope, and expressed as 1+, 2+, 3+ and 4+. Black bars represent strongyle egg-positive cattle. White bars represent strongyle egg-positive goats.

We further focused on analyses of strongyle infection as the parasite was abundantly detected in cattle and goats. The overall infection rate and 95% CI for strongyle infection based on the risk factor categories is shown in Table 3 for cattle and Table 4 for goats. Strongyle egg-positive samples were found in nine of the 11 cattle farms (81.8% herd prevalence, 95% CI = 47.75–96.78%), and in eight of the nine goat farms (88.9% herd prevalence, 95% CI = 50.67–99.42%). Egg burden was determined by semi-quantitative grading of the egg number. As shown in Figure 3, the majority of the strongyle egg-positive cattle (66.7%) were lightly infected (1+), whereas most of the strongyle egg-positive goats (74.3%) were heavily infected (4+).

Results of univariable analysis by Chi-square test showed that production purpose (χ^2^ = 9.873, *p* = 0.002), farm management (χ^2^ = 4.377, *p* = 0.036), deworming interval (χ^2^ = 14.901, *p* < 0.001) and flocks (χ^2^ = 34.499, *p* < 0.001) were significantly associated with the risk of strongyle infection in cattle (Table 3), whereas gender (χ^2^ = 11.456, *p* < 0.001), oral mucous membrane color (χ^2^ = 11.848, *p* = 0.003), production purpose (χ^2^ = 20.052, *p* < 0.001), farm management (χ^2^ = 11.456, *p* < 0.001), deworming interval (χ^2^ = 11.456, *p* < 0.001) and flocks (χ^2^ = 32.197, *p* < 0.001) were significantly associated with the risk of strongyle infection in goats (Table 4).

Multivariable logistic regression analysis (Table 5) further showed that dairy cattle (OR 5.069; 95% CI: 1.461–17.595; *p* = 0.011) were associated with an increased odds of strongyle infection, i.e., dairy cattle were 5.1 times more likely to be infected with strongyle than meat cattle. Male goats (OR 0.189; 95% CI: 0.051–0.702; *p* = 0.013), and dairy goats (OR 0.108; 95% CI: 0.032–0.371; *p* < 0.001) were associated with decreased odds of strongyle infection. Thus, female goats were 5.3 times (95% CI: 1.42–19.52) more likely to be infected with strongyle than male goats; and meat goats were 9.3 times (95% CI: 2.69–31.69) more likely to be infected with strongyle than dairy goats.

### 3.3. Molecular Identification of Gastrointestinal Strongyle in Cattle and Goat Feces

**Figure 4:**
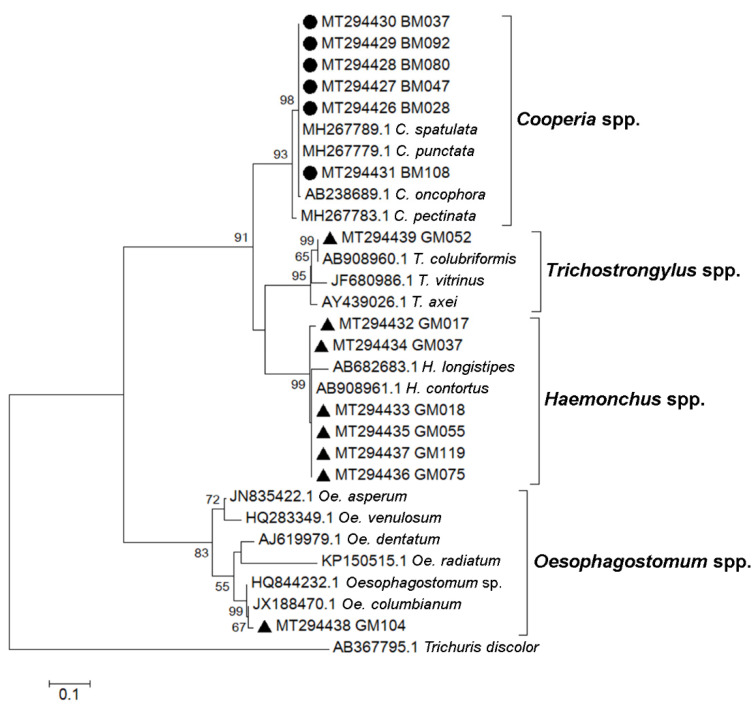
Phylogenetic analysis of ITS2 nucleotide sequences. The tree was constructed using the maximum likelihood method with a bootstrap value of 1000. Black circles represent strongyle sequences from cattle feces, and black triangles represent strongyle sequences from goat feces. Accession numbers of sequences obtained in this study and of reference nematode sequences are indicated. Bootstrap values of ≥50 are shown at the nodes. The bar represents nucleotide substitutions per site. *T. discolor* sequence was used as an outgroup.

To describe the strongyle species circulated in the study area, PCR using primers specific to genomic 5.8S and 28S ribosomal DNA sequences flanking the ITS2 region (primers Strongyle F2/R3) was performed on randomly selected strongyle egg-positive fecal samples (24 samples from cattle and 24 samples from goats). PCR products of the expected sizes were subjected to nucleotide sequencing. The retrieved DNA sequences were analyzed by a BLAST search of the NCBI database, and ITS2 region sequences were compared with reference strongyle sequences, and a phylogenetic tree was then created (Figure 4). We were able to retrieve six strongyle sequences from cattle and eight sequences from goats using nucleotide sequencing. These were from four strongyle genera, *Cooperia* (six sequences from cattle), *Haemonchus* (six sequences from goats), *Oesophagostomum* (one sequence from goats), and *Trichostrongylus* spp. (one sequence from goats). Phylogenetic analysis suggested the presence of *C. spatulata* or *C. punctate* (100% sequence identity), *H. contortus* (>98% sequence identity), *Oe. columbianum* (99% sequence identity), and *T. colubriformis* (100% sequence identity).

During sequence analysis, we noticed that some sequences showed a mix of nucleotides in the sequencing chromatogram, suggesting that a number of sequence variants were co-amplified by the primers. Subsequently, in-house semi-nested PCR primers specific to each of the four strongyle genera were used to detect the species involved in the strongyle coinfections (Table 1). Using nested PCR, strongyle genera can be identified in 79.2% (19/24) of cattle samples and 100% (24/24) of goat samples (Table 6). All four strongyles were detected in both cattle and goat feces. In cattle samples, *Cooperia* (70.8%) was found to be the most abundant, followed by *Trichostrongylus* (45.8%), *Oesophagostomum* (29.2%), and *Haemonchus* spp. (16.7%). In goat samples, *Haemonchus* was detected in all 24 samples (100%), followed by *Trichostrongylus* (91.7%), *Oesophagostomum* (37.5%), and *Cooperia* spp. (37.5%). Our investigation in 24 sampling specimens suggested that the majority of samples from cattle (58.3%) and goats (95.8%) were infected by at least two strongyle species.

## 4. Discussion

Cattle and goats are the main livestock in Thailand and major sources of meat and milk. For the farmers who raise them, infectious diseases that affect animal health and productivity, including bacterial, viral and parasitic (helminth and protozoa) infections, are of concern. It is important to continuously survey for pathogen prevalence, variations, and drug resistance in order to maintain effective intervention strategies within livestock health management. Although a single time point sample collection in a cross-sectional study could limit the sensitivity of parasite detection leading to underestimating of a true parasite prevalence, it provides an accessible and convenient source of up-to-date findings when longitudinal or experimental studies are rarely available [10]. In Thailand, the problem of gastrointestinal helminthic infection in livestock ruminants was well known [6,7,22], but published data to demonstrate its current prevalence was limited. As described above, we performed a cross-sectional epidemiological study of gastrointestinal helminths, especially strongyle nematodes, in cattle and goats in Kanchanaburi Province, which is situated in the western part of Thailand. The area is home to an important part of the livestock industry in Thailand, with many cattle and goat farms. By investigating fecal specimens from 11 cattle farms (*n* = 157) and nine goat farms (*n* = 117), microscopic examination revealed the rates of gastrointestinal helminthic infections in cattle (35.7%) and goats (88%), with the most commonly observed eggs being those of strongyle nematodes. Eggs of *Strongyloides* spp., *Trichuris* spp., *Capillaria* spp., *Paramphistomum* spp., and *Moniezia* spp. were also detected but in lower amounts compared with strongyle eggs.

A survey from Nan Province in the northern part of Thailand, in 2006, showed 61% overall prevalence of gastrointestinal parasitic infections in beef cattle, with rumen flukes (28%) and strongyles (27%) being the most common parasites [6]. In comparison, we reported 35.7% overall GI helminth infection in cattle, with strongyles (28.7%) as a majority followed by the rumen fluke *Paramphistomum* spp. (10.2%). The lower parasite infection rate observed in our study could be due to a sole focus on gastrointestinal helminthic infection, whereas in the previous study both helminths and protozoa were included. Geographical location and climate have also been reported as factors that possibly affect the prevalence and type of parasitic infection in cattle in Thailand [6,22,23]. Kanchanaburi Province is in the western central part of the country whereas Nan Province is in the northern part. The climates in these areas are generally different. For the type of helminths detected, although a comparable infection rate of strongyles was observed, the rate of rumen fluke infection in our study was lower than that observed in Nan Province. A higher prevalence of *Paramphistomum* spp. (25.4%) was also reported in beef cattle from Phayao Province located close to Nan, in 2019 [23]. The difference in fluke prevalence could be due to the distribution of parasite intermediate hosts, which may vary in different regions of Thailand. For goats, there was a limited report of helminth prevalence from Thailand, even less than that reported in cattle. However, the high individual prevalence (88%) and herd prevalence (100%) of gastrointestinal helminth eggs observed in this study concurs with previous findings in Nakhon Pathom Province in western central Thailand in 2012, which reported 100% herd prevalence and 79.5% individual prevalence of intestinal parasites in goats with strongyle nematodes as the most common parasite found in all positive samples [7]. Strongyles have been the most abundant gastrointestinal parasite infecting livestock in studies carried out since 2003 in countries in various regions such as Lao PDR, Malaysia, Philippines, Pakistan, India, Sudan and Germany. The reported prevalence of strongyles ranged from 11.2–36.0% in cattle and 9.3–96.2% in goats [3,4,8,9,10,12,24]. In those studies, *Strongyloides* spp., *Trichuris* spp., *Capillaria* spp., *Paramphistomum* spp., *Fasciola* spp., *Toxocara* spp. and *Moniezia* spp. were also reported. Besides, coccidia protozoa especially *Eimeria* spp. were commonly found in cattle and goat feces, with a wide prevalence that ranged from 2% to 100% [6,10,24,25,26]. Other protozoa including *Entamoeba* spp., *Blastocystis* spp., *Balantidium* spp., *Isospora* spp., *Cryptosporidium* spp. and *Giardia* spp. were also reported [7,26,27]. In our study, we detected the presence of coccidia oocysts in some of the cattle and goat fecal samples, but since it was not our focus, we did not subject them to further analysis. Altogether, these findings demonstrate that, for almost two decades, helminth infections, especially strongyle infections, have been a major problem in livestock in Thailand and other countries. Geographical location and climate could be factors that affect the parasites’ prevalence, together with other animal and farm management factors that differ in different areas. Concerned with the high prevalence and impact of strongyle infection in livestock ruminants, we focused our analysis mainly on strongyle nematodes.

Multivariate analysis showed that the production purpose (dairy or meat) affected the risk of strongyle infection in cattle and goats in Kanchanaburi. Dairy cattle showed a higher risk of strongyle infection than meat cattle, whereas meat goats showed a higher risk of strongyle infection than dairy goats. The opposite results observed in cattle and goats could be explained by the connection of factors subjected to the analysis. In multivariate analysis, if factors were associated with the others, only one factor with the most significance will be presented. While the production purpose was only shown to be significant in multivariate analysis, it was associated with farm management and deworming intervals. All of these three factors were found to be significantly associated with the risk of strongyle infection in both cattle and goats, as shown by the univariate analysis. In fact, it was found in the sampling groups that most of the dairy cattle were housed in intensive management farms with more than six months deworming interval; and most of the meat goats were kept in extensive management farms with more than six months deworming interval, suggesting the important of deworming practice as the factor that determines the risk of strongyle infection. Proper anthelmintic drug treatment using the correct dose, frequency, and route of administration is vital. A single treatment each year was not effective for preventing infection, especially re-infection, which can occur rapidly after treatment [28]. The deworming program of ≥6 months interval implemented in the dairy cattle and meat goat farms was shown to be insufficient. Three months or less deworming intervals have been suggested for efficient reduction of the risk of intestinal parasitic infection in goats by lowering of parasite eggs and larvae present in the animals’ environment [7]. The lack of routine deworming in the dairy cattle may be due to a low level of worm burden that did not cause apparently severe effects to the body condition or health status of the animals, making the farmers unconcerned about the helminthic infection or importance of deworming. Collaborations between the Department of Livestock Development and farmers are needed for the management of effective health care programs that include deworming in livestock farms. An education program should also be available to farm owners to provide basic information about anthelmintic drugs and the effects of parasitic infections on animal production, and to emphasize the importance of health management. Routine surveillance and monitoring of the efficacy of the control programs should be implemented and the infected animals should be promptly treated to reduce the parasitic burden and transmission. Group housing could be another factor that facilitates the parasite transmission, as previously reported [7]. Dairy cattle in the present study were housed intensively in the same pen, meaning that parasitic transmission could occur if there was an infected animal in the herd. The source of infection could be from food and roughage that were used for the animal feeding. Group housing was also used for the meat goats. Although the animals were released to graze freely during the day, they were housed together indoors at night. Free-grazing and sharing of a grazing pasture were shown to be a risk factor for gastrointestinal helminthic infection [6,9]. In addition, it was reported that dairy goat farmers were more concerned about breeding selection and usually practice an appropriate deworming program to maintain good animal health [7]. Sex has been reported as a factor that influences the prevalence of parasitic infection [9,25]. In some reports, males were associated with increasing risk of parasitism due to their free grazing practice compared with females which are kept in stall feeding during pregnancy [29,30]. During pregnancy, female animals could also be prone to parasitic infections due to stress and decreased immunity [9]. In our study, female goats were found to be associated with an increased risk of strongyle infection compared to male goats. However, the number of males (*n* = 18) subjected to the study was less than the females (*n* = 99) and the pregnancy status of the female goats was unknown. Strongyle nematodes are well known to cause gastrointestinal parasitism leading to health problems and decrease in animal productions in small ruminants. Thus, *Haemonchus contortus*, a highly pathogenic blood feeding nematode, has been reported to cause anemia in goats and sheep, which can lead to death in case of heavy infection [14]. Although a high strongyle infection rate and egg burden were detected by microscopy, and *Haemonchus* spp. were detected in all selected egg-positive samples (*n* = 24) from goats by molecular techniques, we did not statistically observe an association between strongyle infection and anemia parameters (oral mucosa color and PCV) by multivariable analysis in the cattle and goats. This was possibly due to mild infections; the animals enrolled in this study were normal. It was shown in the study from Malaysia that PCV of ≤ 22% (anemia) was associated with increased strongyle egg counts of approximately 1000 eggs per gram (EPG) of feces, determined by McMaster egg count technique, compared to approximately 700 EPG in non-anemic goats [25]. In this study, an egg grading (+1 to +4) was performed to semi-quantitatively estimate the strongyle egg burden, not the absolute egg count, therefore we could not relate the infection to egg numbers. Furthermore, oral mucosa color was examined in both cattle and goats for sign of anemia which could be a result of various health conditions such as blood loss anemia, helminth infection or malnutrition. In sheep and goats, FAMACHA eye scoring is practically used for determining anemia status associated with haemonchosis [31]. A pallor of eye mucosa suggests an infection with the blood-sucking *Haemonchus* and further anthelmintic treatment. We note these points and suggest that McMaster egg count technique and FAMACHA eye scoring should be applied in a further study.

Generally, strongyle species cannot be differentiated by examining their eggs; instead, coproculture followed by third-stage larval identification is required, which is time-consuming and requires an experienced examiner. Therefore, relatively rapid, uncomplicated, and cost-effective PCR methods are becoming very useful for identifying and confirming morphological diagnoses [3,4]. PCR and DNA sequencing facilitates helminth identification to the genus or species level and allows the identification of multiple strongyle infections that are difficult to differentiate under the microscope [32]. Although the strongyle species identification may unnecessary for treatment management, the information on strongyle types that infect animals provides an understanding of helminth epidemiology, population biology and anthelmintic treatment effectiveness; all of these are important for planning a helminth control program [4]. In this study, we determined species of strongyle nematodes circulating in cattle and goats in the study area, by DNA sequencing of the ITS2 gene and we identified four genera, *Cooperia* spp., *Haemonchus* spp., *Trichostrongylus* spp. and *Oesophagostomum* spp. These strongyles have been identified, by coproculture or molecular methods, and reported to be common infections of livestock [3,4,8,9,12]. *Haemonchus* spp. were found to be a major strongyle genus infecting goats (25–93% in goat, and 37% in cattle). *Trichostrongylus* spp. (6–76% in goats, and 16–48% in cattle) and *Oesophagostomum* spp. (2–24% in goats, and 6–16% in cattle) were commonly found in goats and cattle. *Cooperia* spp. were mainly found in cattle (prevalence 8–18%). Their prevalence reported in goats was limited (10%). Furthermore, we used in-house-designed semi-nested PCR with primers specific to the ITS2 region to differentiate the four strongyle genera according to variations in the PCR product sizes. Our results were in agreement with the previous studies. *Haemonchus* spp. were abundant in goats (100%), whereas *Cooperia* spp. were mainly found in cattle (70.8% in cattle, and 37.5% in goats). *Trichostrongylus* spp. (91.7% in goats, and 45.8% in cattle) and *Oesophagostomum* spp. (37.5% in goats, and 29.2% in cattle) were common in both animals. Polyparasitism has been commonly observed in cattle and goats. Multiple infections of different helminth or protozoan species, as well as mixed infections of helminths and protozoa have been reported [25,26,27,33,34]. The rates of mixed infection found in previous reports varied from 10% to more than 60%, suggesting that in some settings, polyparasitism was more common than monoparasitism. We observed in our study that 14.3% and 35.9% of helminth egg-positive samples from cattle and goats, respectively, contained more than one kind of helminth egg. The rate of polyparasitism could be higher if protozoa were included in our examination. Using nested PCR, coinfections with different strongyle genera were observed in the majority of the tested samples, suggesting that multiple infection with different strongyle genera in one host was more common than single strongyle infection, in both cattle and goats. Although all strongyle infections can be treated by the same anthelmintic drug, it has become advantageous to know the coinfection status because there were reports showing that coinfections of particular parasites can affect disease severity or animal product quality. Coinfections of *Trichostrongylus* spp. and *Haemonchus* spp., or of these two with other parasites, were shown to impact wool and milk production and health status of small ruminants [35,36]. *Haemonchus* spp. and *Trichostrongylus* spp. coinfection were found to predominate in goats compared to the other coinfections in our study. Compared with *Haemonchus* spp. which are highly concerning because they cause severe disease and anthelminthic resistance, *Trichostrongylus* spp. cause a less severe disease in ruminants [37,38]. However, it was reported in Malaysia to be the second predominant strongyle species after *H. contortus*; and its prevalence was recently found to increase in small ruminants [4]. In our study we found that 91.7% and 45.8% of goat and cattle samples, respectively, were positive for *Trichostrongylus* spp., supporting the suggestion in the previous study that this genus should be viewed with concern as the emerging strongyle species, warranting further monitoring. However, it has to be noted that the samples used for strongyle genus identification in our study were randomly selected from egg-positive samples; therefore, the number reported was not a population prevalence and cannot be absolutely compared with the prevalence obtained in other studies.

## 5. Conclusions

Using microscopic examination, we determined the infection rate of strongyle nematodes and other helminths in cattle and goats in Kanchanaburi Province, Thailand. Molecular identification and sequence analysis of strongyles demonstrated the distribution of the genera *Cooperia*, *Oesophagostomum*, *Haemonchus*, and *Trichostrongylus*. Most of the animals had multiple infections with different strongyle species. The high rate of strongyle infection in dairy cattle and meat goats suggests the ineffectiveness of the current deworming programs in the area. Overall, the results add recent information about helminths, especially strongyle nematode infection, and related risk factors to the field of epidemiological helminthology. It is of great importance to survey and monitor parasitic prevalence and genetic changes to prevent future outbreaks and drug resistance. Molecular methods such as PCR detection have shown their potential for epidemiological study by improving the diagnosis and identification of strongyle species. Education regarding zoonotic diseases and prevention and control should also be provided, especially for those in regular contact with livestock.

## Figures and Tables

**Table 1:** Primer sequences for strongyle detection by semi-nested PCR.

Primer	Sequence (5′-3′)	Region	Product Size (bp) ^1^
Strongyle F2	TGGTGAAATTTTGAACGCATAG	5.8S rRNA	-
Strongyle R3	ATGCTTAAGTTCAGCGGGTA	28S rRNA	324–349
Cooper R	CGAATACTACTATCTCCAACATG	ITS2	293
Haemo R	GTACACTCAAATAGWGGCAACAT	ITS2	227
Oeso R	CTCATCTAGAACGAGGATCACA	ITS2	143
Tricho R	CAATATTTGAYAATGACCATTCG	ITS2	128

^1^ PCR product sizes were estimated from alignment of each primer with representatives of strongyle genera (GenBank: MH267779.1, AB908961.1, HQ844232.1, and AB908960.1).

**Table 2:** Survey of gastrointestinal helminth eggs by microscopic examination.

Helminth Species	No. of Helminth Egg Positive Samples (%)	
	Cattle (*n* = 157)	Goat (*n* = 117)
Strongyle nematodes	45 (28.7)	101 (86.3)
*Strongyloides* spp.	1 (0.6)	18 (15.4)
*Trichuris* spp.	0 (0)	7 (6)
*Capillaria* spp.	1 (0.6)	5 (4.3)
*Paramphistomum* spp.	16 (10.2)	9 (7.7)
*Moniezia* spp.	1 (0.6)	5 (4.3)
Total ^1^	56 (35.7)	103 (88)

^1^ Total number of fecal samples that were helminth egg-positive. Some samples contained more than one type of egg.

**Table 3:** The overall infection rate and univariable analysis for risk factors of strongyle infection in cattle.

Factor	Number	Infection Rate (%)	95% CI	χ^2^	*p*-Value
Gender					
Male	4	1 (25.0)	4.55–69.93	0.0269	0.8697
Female	153	44 (28.8)	22.17–36.38	-	-
Age					
Young	0	0 (0)	-	-	-
Adult	157	45 (28.7)	22.16–36.17	-	-
Body condition score					
Fat	22	7 (31.8)	16.36–52.68	0.481	0.786
Average	117	34 (29.1)	21.60–37.84	-	-
Thin	18	4 (22.2)	9.01–45.21	-	-
Oral mucosa color					
Pink	85	29 (34.1)	24.92–44.68	2.886	0.2361
Pale pink	69	15 (21.7)	13.64–32.81	-	-
Pale	3	1 (33.3)	6.14–79.23	-	-
PCV categories					
Non-anemic	131	40 (30.5)	23.29–38.88	1.356	0.244
Anemic	26	5 (19.2)	8.51–37.87	-	-
Production purpose					
Dairy	125	43 (34.4)	26.64–43.08	9.873	0.002 *
Meat	32	2 (6.3)	1.73–20.14	-	-
Management					
Intensive	141	44 (31.2)	24.14–39.26	4.377	0.036 *
Extensive	16	1 (6.3)	0.28–8.71	-	-
Deworming interval					
≤6 months	30	0 (0)	0.00–11.35	14.901	<0.001 *
>6 months	127	45 (35.4)	27.65–44.06	-	-
^a^ Flocks					
A	16	1 (6.3)	1.11–28.32	34.499	<0.001 *
B	1	1 (100)	20.65–100.00	-	-
C	14	6 (42.9)	21.38–67.41	-	-
D	15	7 (46.7)	24.81–69.88	-	-
E	15	2 (13.3)	3.73–37.88	-	-
F	13	8 (61.5)	35.52–82.29	-	-
G	13	6 (46.2)	23.21–70.86	-	-
H	15	0 (0)	0.00–20.39	-	-
I	20	6 (30)	14.55–51.89	-	-
J	20	8 (40)	21.88–61.34	-	-
K	15	0 (0)	0.00–20.39	-	-
Overall	157	45 (28.7)	22.16–36.18	-	-

* Statistically significant at *p* < 0.05. ^a^ Letters A–K represent cattle flocks.

**Table 4:** The overall infection rate and univariable analysis for risk factors of strongyle infection in goats.

Factor	Number	Infection Rate (%)	95% CI	χ^2^	*p*-Value
Gender					
Male	18	11 (61.1)	38.61–79.69	11.456	<0.001 *
Female	99	90 (90.9)	83.61–95.14	-	-
Age					
Young	5	4 (80)	37.55–96.37	0.177	0.674
Adult	112	97 (86.6)	79.07–91.71	-	-
Body condition score					
Fat	15	12 (80)	54.81–92.95	4.006	0.134
Average	82	69 (84.1)	74.74–90.49	-	-
Thin	20	20 (100)	83.88–100.00	-	-
Oral mucosa color					
Pink	37	26 (70.3)	54.21–82.51	11.848	0.003 *
Pale pink	79	74 (93.7)	86.02–97.26	-	-
Pale	1	1 (100)	20.65–100.00	-	-
PCV categories					
Non-anemic	98	83 (84.7)	76.27–90.49	1.3597	0.243
Anemic	19	18 (94.7)	75.36–99.06	-	-
Production purpose				-	-
Dairy	20	11 (55)	34.20–74.18	20.052	<0.001 *
Meat	97	90 (92.8)	85.84–96.46	-	-
Management					
Intensive	18	11 (61.1)	38.61–79.69	11.456	<0.001 *
Extensive	99	90 (90.9)	83.61–95.14	-	-
Deworming interval					
≤6 months	18	11 (61.1)	38.61–79.69	11.456	<0.001 *
>6 months	99	90 (90.9)	83.61–95.14	-	-
^a^ Flocks					
a	18	11 (61.1)	38.61–79.69	32.197	<0.001 *
b	15	14 (93.3)	70.18–98.81	-	-
c	15	14 (93.3)	70.18–98.81	-	-
d	15	11 (73.3)	48.04–89.10	-	-
e	19	19 (100)	83.18–100.00	-	-
f	2	0 (0)	0.00–65.76	-	-
g	15	14 (93.3)	70.18–98.81	-	-
h	3	3 (100)	43.85–100.00	-	-
i	15	15 (100)	79.61–100.00	-	-
Overall	117	101 (86.3)	78.93–91.40	-	-

* Statistically significant at *p* < 0.05. ^a^ Letters a–i represent goat flocks.

**Table 5:** Multiple logistic regression of risk factors associated with strongyle infection in cattle and goats.

Risk Factor	Coefficient	SE	z-Score	*p*-Value	AOR	95% CI
Cattle						
Production purpose	1.6232	0.6349	2.556	0.011	5.069	1.461–17.595
Goat						
Gender	−1.6625	0.6679	−2.489	0.013	0.189	0.051–0.702
Production purpose	−2.2241	0.6286	−3.538	<0.001	0.108	0.032–0.371

**Table 6:** Strongyle genera identified in this study using semi-nested PCR.

Strongyle Infection	No. of Strongyle Detected (%)	
	Cattle (*n* = 24)	Goat (*n* = 24)
Strongyle detection by PCR	19 (79.2)	24 (100)
*Cooperia* spp.	17 (70.8)	9 (37.5)
*Haemonchus* spp.	4 (16.7)	24 (100)
*Oesophagostomum* spp.	7 (29.2)	9 (37.5)
*Trichostrongylus* spp.	11 (45.8)	22 (91.7)
Single infection	5 (20.8)	1 (4.2)
*Cooperia* spp.	4 (16.7)	-
*Haemonchus* spp.	-	1 (4.2)
*Oesophagostomum* spp.	1 (4.2)	-
Coinfection	14 (58.3)	23 (95.8)
C + H	2 (8.3)	1 (4.2)
C + O	1 (4.2)	-
C + T	4 (16.7)	-
H + T	-	9 (37.5)
O + T	1 (4.2)	-
C + H + T	2 (8.3)	4 (16.7)
C + O + T	4 (16.7)	-
H + O + T	-	5 (20.8)
C + H + O + T	-	4 (16.7)

C, *Cooperia* spp.; H, *Haemonchus* spp.; O, *Oesophagostomum* spp.; T, *Trichostrongylus* spp.

## Data Availability

Nucleotide sequences of partial ITS2 have been submitted to the GenBank database. Accession numbers are provided in the materials and methods section, and in Figure 4.

## Supplementary Materials

The following are available online at https://www.mdpi.com/article/10.3390/vetsci8120324/s1, Figure S1: Alignments of strongyle genus-specific primers with sequences of *Cooperia* spp. (C), *Haemonchus* spp. (H), *Oesophagostomum* spp. (O), and *Trichostrongylus* spp. (T), Figure S2: Specific amplification of ITS-2 gene fragments with strongyle genus-specific primers.
