# Supplementary material for: Helminth Infections in Cattle and Goats in Kanchanaburi, Thailand, with Focus on Strongyle Nematode Infections
Source: Vet Sci. 2021 Dec 12;8(12):324. doi: 10.3390/vetsci8120324 (PMC8709319; doi:10.3390/vetsci8120324)
Supplement: Supplementary file 1 [file vetsci-08-00324-s001.zip › vetsci-1455494-supplementary.pdf]

```
                                           10        20        30        40        50        60        70        80        90       100
                                   ....|....|....|....|....|....|....|....|....|....|....|....|....|....|....|....|....|....|....|....|
MH267779.1 C. punctata             TGGTGAAATTTTGAACGCATAGCGCCGTTGGGTTTTCCCTTCGGCACGTCTGGTTCAGGGTTGTTAACGATATACTACAGTGTGGCTAACGTTTTAACAC
AB908961.1 H. contortus            TGGTGAAATTTTGAACGCATAGCGCCGTTGGGTTTTCCCTTCGGCACGTCTGGTTCAGGGTTGTTAACCATATACTACAATGTGGCTAA--TTTCAACAT
HQ844232.1 Oesophagostomum sp.     TGGTGAAATTTTGAACGCATAGCGCCGTTGGGTTTTCCCTTCGGCACGTCTGGTTCAGGGTTGTATAT-AAATACTACAGTGTGGCTTG----TGACAC
AB908960.1 T. colubriformis       TGGTGAAATTTTGAACGCATAGCGCCGTTGGGTTTTCCCTTCGGCACGTCTGGTTCAGGGTTGTTAATGAATTTCTACAGTGTGGCTAA--CTCTAACAC
Strongyle F2                       TGGTGAAATTTTGAACGCATAG------------------------------------------------------------------------------

                                          110       120       130       140       150       160       170       180       190       200
                                   ....|....|....|....|....|....|....|....|....|....|....|....|....|....|....|....|....|....|....|....|
MH267779.1 C. punctata             TGTTTGTCTAATGGCATTTGTCTACACCTGTTTTGACAAATCCCGTTATAGTTCAAGAATA-ACATATGCAACATAATGTTCATATAGTAATG---TGAA
AB908961.1 H. contortus            TGTTTGTCAAATGGCATTTGTCT-------TTTAGACAATTCCCATTTCAGTTCAAGAACATATACATGCAACGTGATGT---TATGAAATTG---T-AA
HQ844232.1 Oesophagostomum sp.     TGTTTGTCGAACGATGCTTGCAT-------TTAATGTGATCCTCGTTCTAGATGAGAAATACAT---TGCAACATGCACT--ATGGATCAATC---CAT
AB908960.1 T. colubriformis       TGTTTGTCGAATGGTCATTGTCAAA---TATTGTGATGATTCCCATTTCAGTTCAAGAATA-ATACATGCAACATGATGTTAATGTTGTAATGACATTAA
Oeso R                             ----------------------------------TGTGATCCTCGTTCTAGATGAG--------------------------------------------
Tricho R                           -------CGAATGGTCATTGTCAAA---TATTG-------------------------------------------------------------------

                                          210       220       230       240       250       260       270       280       290       300
                                   ....|....|....|....|....|....|....|....|....|....|....|....|....|....|....|....|....|....|....|....|
MH267779.1 C. punctata             CATTTCTGAATGATATGAATCTATTATCACTGTTTAAATGTACTCAATGAATAT-GAGATTGATTTAAACAGGGACATGT-TGGAGATAGTAGTATTCGT
AB908961.1 H. contortus            CATTCCTGAATGATATGAACATGTTGCCACTATTTGAGTGTACTCAGCGAATATTGAGATTGACTTAGATAGTGACTTGTATGGCGACGATGTTCTT--T
HQ844232.1 Oesophagostomum sp.     GGTGCACGGATGTCGTGACCTCGTTGTCACTGTCAAAGCGT--TTAGCGACTA---AGAATGCTTTGGC--GGGGCCTGTATGACAACTGTGGTTCC--A
AB908960.1 T. colubriformis       TGTTCCTGTATGATGTGAACGTGTTGTTACTGTTTGAATGTACTCAGTGAATTT-GAGATTGATTTAAACAGGGACATGTATAACAATAATGTTCAA--T
Cooper R                           ---------------------------------------------------------------------------CATGT-TGGAGATAGTAGTATTCG-
Haemon R                           --------------------ATGTTGCCWCTATTTGAGTGTAC---------------------------------------------------------

                                          310       320       330       340       350
                                   ....|....|....|....|....|....|....|....|....|....|....|..
MH267779.1 C. punctata             TATCATTTGTAT--TGCAACCTGAGCTCAGGCGTGATTACCCGCTGAACTTAAGCAT
AB908961.1 H. contortus            TATCATTTGTATAATGCAACCTGAGCTCAGGCGTGA-TACCCGCTGAACTTAAGCAT
HQ844232.1 Oesophagostomum sp.     TGTCATTTGCAA--TGCAACCTGAGCTCAGTCGTGATTACCCGCTGAACTTAAGCAT
AB908960.1 T. colubriformis       TATCATTTGTAT--TGCAACCTGAACTCAGGCGTGATTACCCGCTGAACTTAAGCAT
Strongyle R3                       -------------------------------------TACCCGCTGAACTTAAGCAT
```

Product sizes
F2/R3 C-349 bp, H-338 bp, O-324 bp, T-346 bp
F2/Cooper R-293 bp
F2/Haemon R 227 bp
F2/Oeso R 143 bp
F2/Tricho R 128 bp

Figure S1 Alignments of strongyle genus-specific primers with sequences of *Cooperia* spp. (C), *Haemonchus* spp. (H), *Oesophagostomum* spp. (O), and *Trichostrongylus* spp. (T).

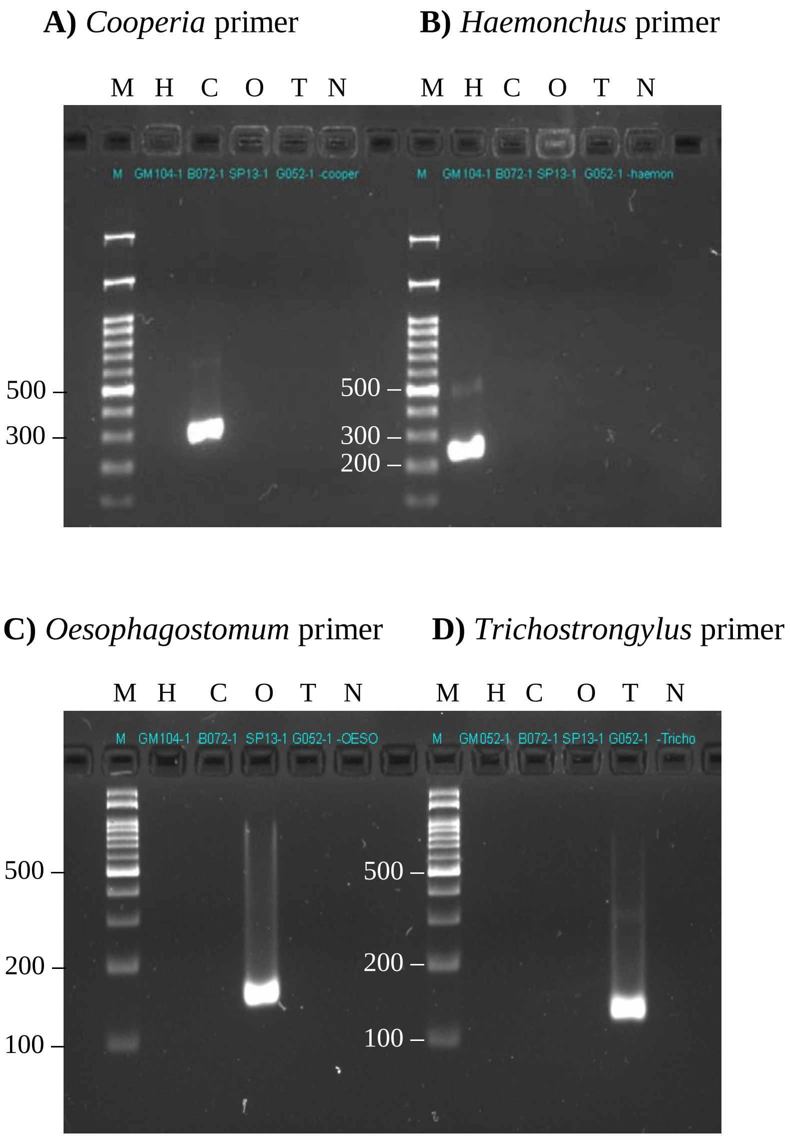

Figure S2 Specific amplification of ITS-2 gene fragments with strongyle genus-specific primers. ITS-2 DNA fragments of 4 strongyles were amplified using primers Strongyle F2/R3 and cloned into pGEM-T Easy plasmid. The DNA fragments in the plasmid were subjected to DNA sequencing and BLAST analysis to confirm the strongyle genus [*Haemonchus* spp. (H), *Cooperia* spp. (C), *Oesophagostomum* spp. (O), and *Trichostrongylus* spp. (T)]. The plasmids were used in PCR reaction with a specific primer for each strongyle. Gel electrophoresis showed specific amplifications of the strongyle ITS-2 using specific primers for *Cooperia* spp. (Figure S2 A), *Haemonchus* spp. (Figure S2 B), *Oesophagostomum* spp. (Figure S2 C), and *Trichostrongylus* spp. (Figure S2 D) with PCR product sizes approximately 290, 230, 140 and 130 bp, respectively. M; 100 bp marker, N; negative control.
